# Engineered membraneless organelles in *Corynebacterium glutamicum* for enhanced indigoidine biosynthesis and antimicrobial peptide production

**DOI:** 10.1016/j.synbio.2025.08.001

**Published:** 2025-08-05

**Authors:** Manman Sun, Yimeng Zhao, Rodrigo Ledesma-Amaro, Jin Gao, Xiuxia Liu, Zhonghu Bai, Alex Xiong Gao, Peng Wang

**Affiliations:** a Key Laboratory of High Magnetic Field and Ion Beam Physical Biology, Hefei Institutes of Physical Science, Chinese Academy of Sciences, Hefei, 230031, China; b Institute of Hefei Artificial Intelligence Breeding Accelerator, Hefei, 230000, China; c School of Food and Nutrition, Anhui Agricultural University, Hefei, 230036, China; d Department of Bioengineering and Imperial College Centre for Synthetic Biology, Imperial College London, London, SW7 2AZ, UK; e Department of Neurobiology and Cellular Biology, Xuzhou Medical University, Xuzhou, 221004, Jiangsu, China; f National Engineering Research Center of Cereal Fermentation and Food Biomanufacturing, Jiangnan University, Wuxi, 214112, China; g Division of Life Science, The Hong Kong University of Science and Technology, Hong Kong, 999077, China

**Keywords:** liquid−liquid phase separation, *Corynebacterium glutamicum*, Membraneless compartment, Indigoidine, Antimicrobial peptides

## Abstract

Liquid-liquid phase separation (LLPS)-driven membraneless organelles (MLOs) have been employed to enhance metabolic efficiency in various microbial cell factories. However, their application in the industrial bacterium *Corynebacterium glutamicum* has not been explored. Here, we report the formation of liquid protein condensates in *C. glutamicum* using the RGG domain of *Caenorhabditis elegans* LAF-1. We optimized conditions for condensate formation, including the pre-induction period, inducer concentration, and cultivation temperature. Using the indigoidine biosynthesis pathway as a model, we demonstrated that LLPS-mediated MLOs enhanced indigoidine production. Furthermore, we applied these MLOs to modulate the toxicity of antimicrobial peptides (AMPs) to host cells, facilitating the expression of AMPs, including melittin and lactoferricin B. These findings provide insights into MLOs engineering in *C. glutamicum* and suggest broader applications of LLPS-mediated systems in industrial biotechnology.

## Graphical abstract

**Figure:**
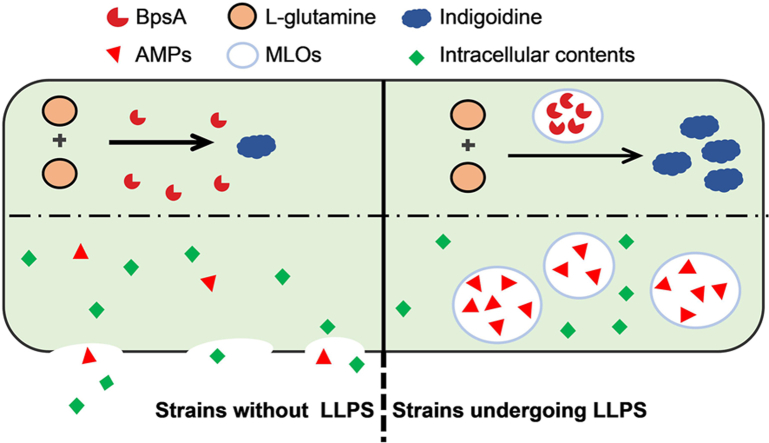

## 1 Introduction

Synthetic biology utilizes engineered biological systems as cellular factories for the sustainable production of valuable products, including chemicals, biofuels, nutraceuticals, and pharmaceuticals [1]. However, these cell factories often face challenges such as low titers of target metabolites and cytotoxicity caused by metabolic intermediates or products [2,3]. Traditional strategies, including fine-tuning gene expression using genetic elements of varying strengths or optimizing cultivation conditions, have been widely applied to overcome these challenges [4,5].

Recently, the development of functional membraneless organelles (MLOs) through liquid-liquid phase separation (LLPS) has emerged as a versatile and promising strategy to address these challenges [[6], [7], [8], [9]]. By compartmentalizing specific enzymes and substrates within the cell, MLOs can enhance reaction rates and increase the production titers of target products. For example, Yu et al. constructed membraneless compartments in *Bacillus subtilis* and localized key enzymes of the 2′-fucosyllactose (2′-FL) biosynthetic pathway to the condensates, resulting in a 2.2-fold improvement in 2′-FL titer [7]. Beyond enhancing metabolic efficiency, these MLOs have been utilized to control enzyme activities, regulate cellular behavior, improve gene-editing efficiency, and prevent protein aggregation and degradation [[10], [11], [12], [13]]. Theoretically, this design could also be applied to isolate or mitigate the toxic effects of proteins on host cells; however, this has yet to be demonstrated.

The construction of synthetic MLOs typically relies on the fusion of intrinsically disordered proteins (IDPs) with low-complexity amino acid sequences [14]. These IDPs form dynamic and reversible droplet-like structures through weak intermolecular forces such as electrostatic and aromatic interactions [15]. Currently, various IDPs, including FUS protein, mini-spidroin (NT2RepCT), and elastin-like polypeptides (ELPs), have been successfully utilized to generate phase-separated protein condensates in bacteria, yeast, and mammalian cells [6,16,17]. Furthermore, researchers have designed artificial IDPs (A-IDPs) and synthetic IDPs (SIDPs) to expand the toolkit for constructing such organelles [7,10]. Among these, the RGG domain of LAF-1 from *Caenorhabditis elegans* stands out as a powerful molecular tool for constructing artificial MLOs due to its unique phase separation properties in both in vitro and in vivo settings [8,12,18].

Although the application of MLOs has shown great promise in model hosts such as *Escherichia coli*, *B. subtilis*, and yeast, further exploring its application in other industrial chassis is also necessary [6,7,16]. *Corynebacterium glutamicum*, a Gram-positive bacterium isolated from soil, was initially used for amino acid and organic acid production [19]. Over the past decade, it has also been demonstrated as a promising cell factory for recombinant protein expression due to its endotoxin-free nature, superior secretion capability, low extracellular protease activity, and generally recognized as safe (GRAS) status [20,21]. With the continuous development of gene editing tools, efficient expression elements, and deeper insights into its gene regulatory networks, protein secretion mechanisms, and metabolic physiology, *C. glutamicum* is emerging as a multifunctional biosynthetic platform with broad industrial potential [[22], [23], [24], [25], [26], [27]].

In this study, we aimed to construct MLOs in *C. glutamicum* using the RGG domain and investigate the effects of cultivation conditions on condensate formation. We also assessed the potential of MLOs to enhance indigoidine synthesis efficiency and mitigate the cytotoxicity of antimicrobial peptides (AMPs) to host cells. Our findings provide valuable insights into the construction of MLOs in *C. glutamicum* and expand the application scenarios of LLPS-mediated MLOs in industrial biotechnology.

## 2 Results

### 2.1 Design and demonstration of membraneless compartments in *C. glutamicum*

**Fig. 1:**
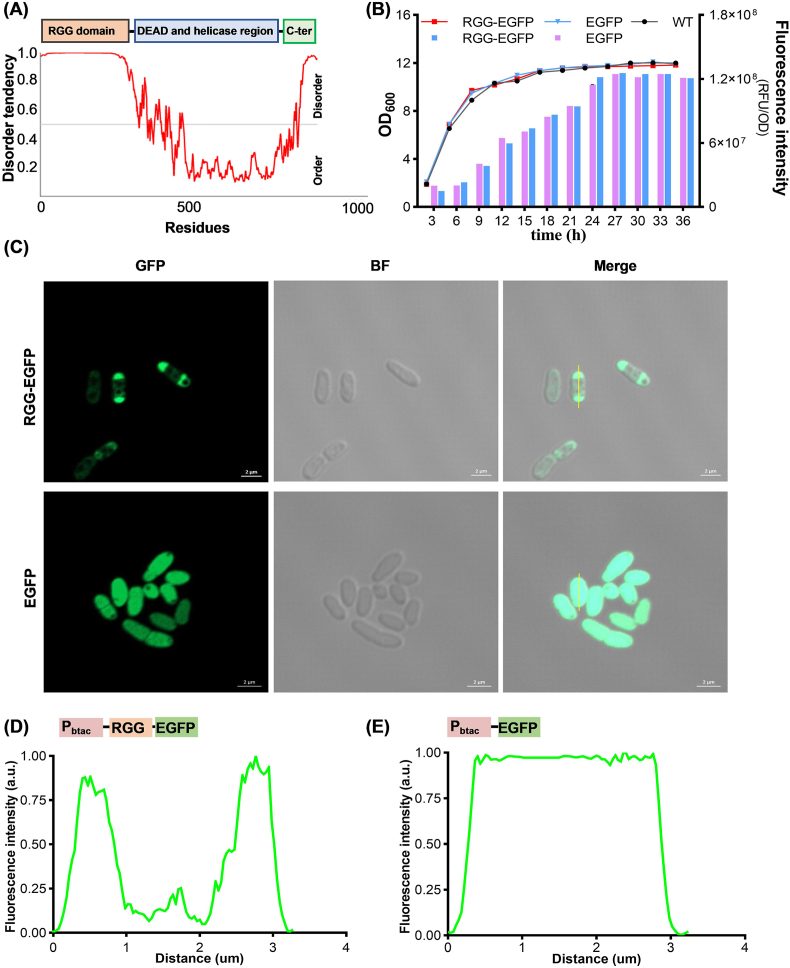
Expression of a single RGG domain in *C. glutamicum* generates intracellular compartments. (A) The intrinsic disorder propensity analysis of LAF-1. Disordered regions were defined using the established threshold criterion, with scores exceeding 0.5 denoting disordered conformational states. (B) Analysis of fluorescence intensity and cell growth of recombinant strains at different time points. Line: bacterial growth, bar: fluorescence intensity. (C) Confocal microscope images of strains expressing the RGG-EGFP and EGFP, BF represents the bright field. (D,E) Fluorescence distribution analysis of strains expressing the RGG-EGFP and EGFP using ImageJ software.

Previous studies have demonstrated that the intrinsically disordered RGG domain derived from the protein LAF-1 serves as an excellent scaffold for constructing MLOs both in vivo and in vitro [8,12,18]. The RGG domain consists of 168 amino acid residues, and its disorder tendency was predicted using the AIUPred (https://iupred.elte.hu/) (Fig. 1A), which revealed a high degree of intrinsic disorder. In this study, we utilized the RGG domain to construct membraneless compartments in *C. glutamicum*. To evaluate the feasibility of RGG domain-mediated MLOs formation, the RGG sequence was codon-optimized and fused to the N-terminus of enhanced green fluorescent protein (EGFP). The fusion sequence was subsequently cloned into our previously developed enhanced bicistronic expression vector Pbtac-HT11, using *Hin*dIII and *Eco*RI restriction sites [27] (Fig. S1). SDS-PAGE analysis confirmed successful expression of the RGG-EGFP fusion protein at the expected molecular weight (Fig. S2A). An additional band around 38 kDa, likely due to protein degradation, was also observed in the RGG-EGFP expressing strain. Fluorescence intensity measurements and cell growth curve analyses further indicated that the fusion of RGG did not impair EGFP expression and cell growth (Fig. 1B).

Fluorescence microscopy and fluorescence distribution analysis revealed that EGFP fluorescence was evenly dispersed throughout the cytoplasm in cells expressing EGFP alone. In contrast, the RGG-EGFP fusion protein was predominantly localized to the cell poles (Fig. 1C–E). Interestingly, while all cells expressed EGFP, heterogeneity in fluorescence intensity was observed among cells expressing both EGFP and RGG-EGFP. This heterogeneity may result from multiple factors, including variations in cell growth stages, plasmid copy number, and/or protein degradation rates [[28], [29], [30]]. Despite this variation, these morphological features are highly consistent with previously reported MLOs in other microbial systems, suggesting that the RGG domain can effectively drive phase separation in *C. glutamicum* [6,7,16].

### 2.2 Characterization of RGG-mediated membraneless compartments

**Fig. 2:**
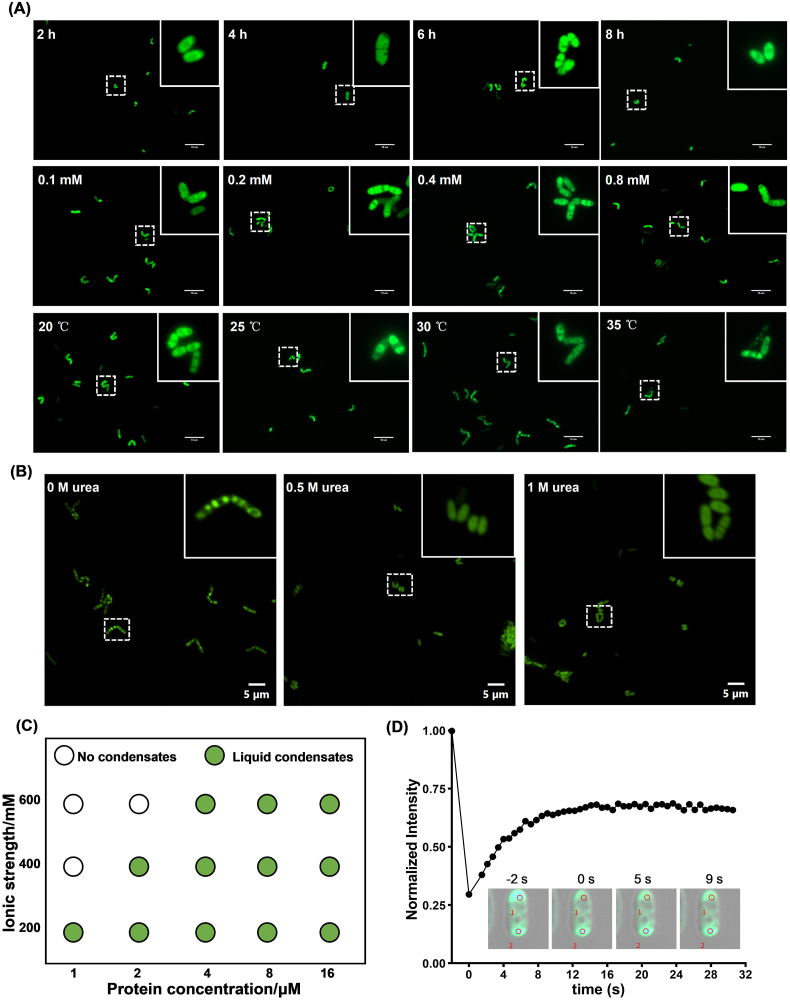
Optimization of cultivation conditions and characterization analysis of RGG-mediated MLOs. (A) Effects of different pre-induction periods, Isopropyl β-d-1-thiogalactopyranoside (IPTG) concentrations, and cultivation temperatures on the formation of intracellular protein condensates. (B) Effects of urea addition on the formation of intracellular protein condensates. (C) A regime diagram illustrates the formation of RGG-EGFP condensates at different protein and salt concentrations. (D) Fluorescence recovery analysis. The inset shows representative images of photobleaching. 1: region of interest 1 (ROI 1, bleached region), 2: region of interest 2 (ROI 2, control region). The average fluorescence intensity in ROI 1 before bleaching was set as the baseline value (defined as 1).

Fusion expression with a single RGG domain in *C. glutamicum* successfully induced MLOs formation via LLPS. However, occasional morphological abnormalities, such as cell enlargement and distortion, were observed (Fig. S2B and S2C). To address this, we systematically optimized the culture conditions to enhance LLPS efficiency and minimize cellular stress. Specifically, we investigated the impact of pre-induction period, inducer concentration, and cultivation temperature on MLOs formation and fluorescence intensity. The resulting data on EGFP intensity and intracellular distribution under various conditions are shown in Fig. 2A and Fig. S3. Ultimately, we determined the optimal conditions to be pre-culturing at 30 °C for 6 h, followed by induction with 0.2 mM IPTG at 25 °C for 30 h, which resulted in the most effective phase separation. Previous studies have demonstrated that increasing the number of RGG repeats enhances LLPS propensity compared to a single RGG domain [12,31]. In this study, we constructed the RGG-RGG-EGFP expressing strain. However, in *C. glutamicum*, the dual-RGG system exhibited inferior performance compared to the single-RGG construct, as evidenced by reduced fluorescence intensity and less pronounced fluorescent polarization in Fig. S4A–S4D and Fig. S4G.

To further characterize the properties of RGG-mediated MLOs, we purified the RGG-EGFP protein using nickel affinity chromatography and investigated its behavior under various conditions. In vitro phase separation assays revealed that LLPS-driven droplet formation occurred at protein concentrations as low as 1 μM (200 mM NaCl, pH = 7.4, 25 °C) (Fig. S4E and S4F). Lower ionic strength and higher protein concentrations are more conducive to the formation of liquid condensates, while higher ionic strength inhibits condensate formation (Fig. 2C). Given that low concentrations of urea are known to disrupt liquid-like condensates, we next examined the effect of urea treatment on phase separation in vivo. The result showed that when RGG-EGFP-expressing *C. glutamicum* cells were exposed to either 0.5 M or 1 M urea, the intracellular condensates were disrupted, confirming that the condensates formed in *C. glutamicum* are liquid-like in nature (Fig. 2B) [7,32]. To further evaluate the fluidity of these condensates, fluorescence recovery after photobleaching (FRAP) was performed on intracellular RGG-EGFP droplets. Following photobleaching, fluorescence recovered to approximately 65 % of its original intensity within 9 s (Fig. 2D). Confirming that RGG-mediated condensates exhibit typical liquid-like characteristics with high fluidity and dynamic exchange properties.

**Fig. 3:**
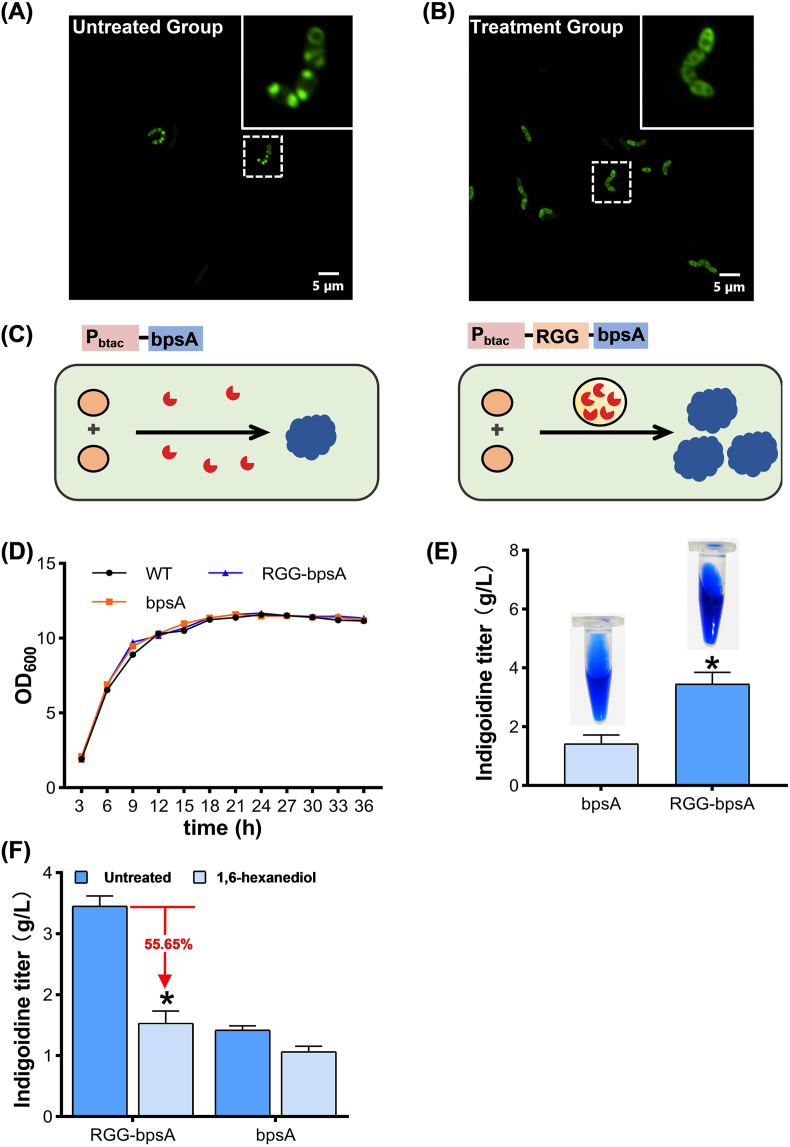
Application of RGG-mediated MLOs in enhancing indigoidine biosynthesis. (A,B) Effects of 4 % (v/v) 1,6-hexanediol treatment on synthetic cellular condensates. (C) Schematic diagram of indigoidine synthesis pathway in *C. glutamicum*. RGG-mediated MLOs can increase local enzyme concentration to enhance indigoidine synthesis. Circles represent l-glutamine, red shapes represent bpsA enzyme, and blue shapes represent indigoidine. (D) Effects of RGG-bpsA and bpsA expression on bacterial growth. (E) Effects of RGG-mediated MLOs on indigoidine synthesis, in mean ± SEM, n = 3. (∗) *p* < 0.05. (F) Effects of 4 % (v/v) 1,6-hexanediol treatment on indigoidine production. In mean ± SEM, n = 3. (∗) *p* < 0.05.

Finally, we explored the role of weak multivalent interactions in condensate formation by treating cells with 1,6-hexanediol, a well-established LLPS disruptor [33,34]. Various concentrations of 1,6-hexanediol (2 %, 3 %, 4 %, and 5 % v/v) were tested to evaluate their effects on cell growth and MLOs formation. Among them, 4 % (v/v) 1,6-hexanediol was selected, as it caused a moderate reduction in cell growth (∼10 %) but significantly diminished EGFP polarization at the cell poles (Fig. 3A and B, and Fig. S5). These findings further confirm that weak, reversible interactions are critical for LLPS and condensate formation. Collectively, our study demonstrates that RGG-mediated compartments in *C. glutamicum* are liquid-like in nature, highly dynamic, and rely on multivalent interactions to drive their formation via LLPS.

### 2.3 Spatial organization of indigoidine biosynthetic pathway via phase-separated compartmentalization

Having established cellular condensate in *C. glutamicum*, we next explored the utility of LLPS-mediated metabolic compartmentalization for improving biosynthetic efficiency. Indigoidine, an ancient dye historically used in textile dyeing, was chosen as a model biosynthetic product. *C. glutamicum* serves as an ideal chassis for indigoidine biosynthesis due to its native capacity to overproduce l-glutamine precursors and its endogenous phosphopantetheinyl transferase (PPTase) activity, requiring only the heterologous expression of the bpsA gene, which encodes indigoidine synthase [35]. In this study, bpsA was expressed either alone or fused with the RGG domain to evaluate the impact of LLPS-mediated compartmentalization (Fig. S1).

To determine the optimal detection wavelength for indigoidine, its absorption spectrum was measured via full-wavelength scanning (500–750 nm). A characteristic absorption peak at 620 nm was identified (Fig. S6A), which was subsequently used for quantitative analysis. A standard curve for indigoidine quantification was established based on its absorbance at 620 nm (Fig. S6B). Results showed that RGG-bpsA expression did not affect bacterial growth but significantly increased the indigoidine titer, reaching 3.45 g/L, a 2.43-fold increase compared to the control expressing bpsA alone (Fig. 3C–E). Furthermore, the observed enhancement in indigoidine production was not attributable to changes in bpsA expression level, as the protein expression level of RGG-fused bpsA was comparable to that of the non-fused control (Fig. S6C and S6D). Time-course measurements of indigoidine production further confirmed the superior performance of the RGG-bpsA strain, consistently yielding higher titers than the bpsA strain at all time points (Fig. S6E).

To investigate whether the enhanced indigoidine biosynthesis is linked to RGG-mediated MLOs formation, we treated both bpsA and RGG-bpsA strains with 4 % (v/v) 1,6-hexanediol, a chemical known to disrupt LLPS [33,34]. While 1,6-hexanediol moderately impacted bacterial growth (approximately 10 % reduction), it did not significantly alter the expression levels of bpsA or RGG-bpsA (Fig. S6C and S6D). Upon treatment with 4 % (v/v) 1,6-hexanediol, indigoidine production in the LLPS group (RGG-bpsA) decreased significantly (55.65 ± 1.85 %) (Fig. 3F). The non-LLPS control group (bpsA) also exhibited a reduction of 25 %, likely due to the physiological effects of 1,6-hexanediol treatment on cellular metabolism. Notably, even after 1,6-hexanediol treatment, the LLPS group retained 43.66 % higher production than the non-LLPS group. This suggests that 4 % (v/v) 1,6-hexanediol did not fully abolish phase separation, consistent with the partial reduction in EGFP fluorescence polarization observed in Fig. 3A and B. Collectively, these results confirm that RGG-mediated MLOs enhance the biosynthetic efficiency of indigoidine in *C. glutamicum*, highlighting the potential of phase separation engineering as a strategy for metabolic optimization.

### 2.4 Enhanced AMPs production through membraneless organelle-mediated toxin shielding

**Fig. 4:**
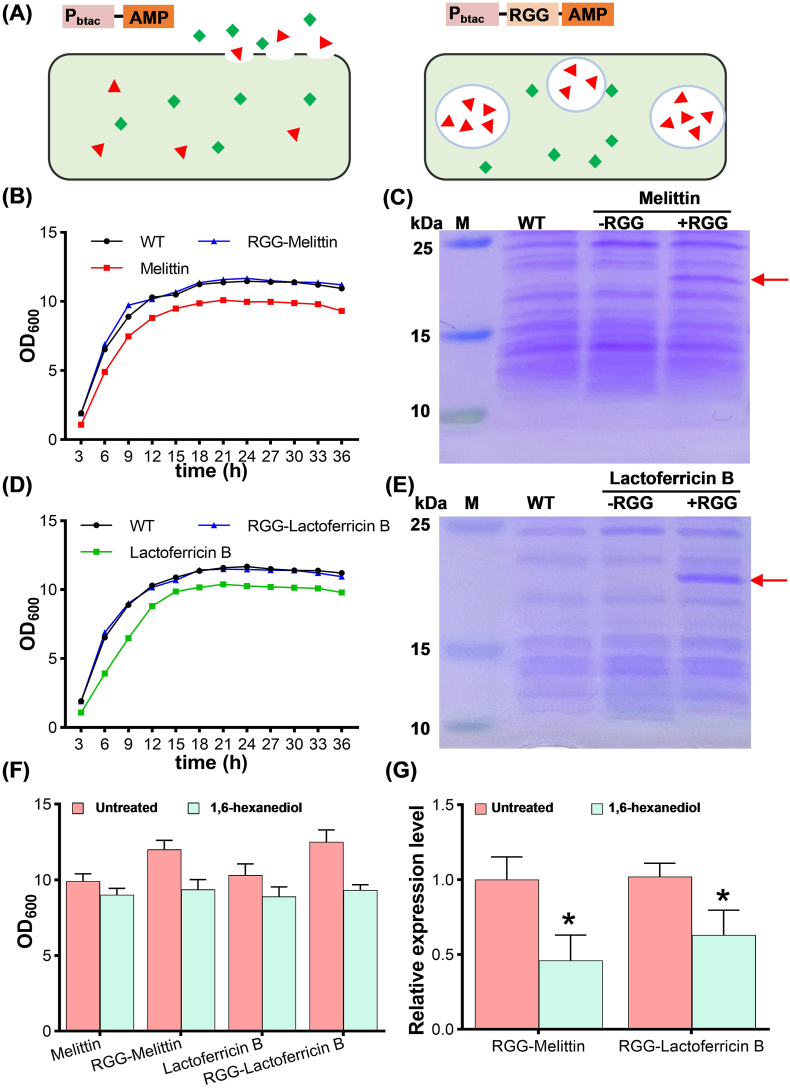
Application of RGG-mediated MLOs in enhancing AMPs production. (A) Schematic diagram of MLOs enhancing AMPs production via toxin shielding. Red triangles represent AMPs, green diamonds denote intracellular contents, and circles represent LLPS-mediated MLOs. The left one represents cell damage and content leakage, while the right one shows MLOs isolating the toxicity of AMPs from the cells. (B) Effects of RGG-Melittin and Melittin expression on bacterial growth. (C) SDS-PAGE analysis of RGG-Melittin expression. Line M represents Marker. WT represents the control that does not express AMPs. (D) Effects of RGG-Lactoferricin B and Lactoferricin B expression on bacterial growth. (E) SDS-PAGE analysis of RGG-Lactoferricin B expression. Line M represents Marker. WT represents the control that does not express AMPs. (F) Effects of 1,6-hexanediol treatment on cell growth. (G) Effects of 1,6-hexanediol treatment on AMPs expression levels. The AMPs expression level in the untreated RGG-AMPs expressing strain was set as a baseline and defined as 1, significant differences (∗) were observed at *p* < 0.05 compared to the untreated RGG-AMPs group.

LLPS-mediated MLOs may also offer a promising strategy to mitigate protein toxicity by physically sequestering toxic proteins away from host cellular components. Antimicrobial peptides (AMPs), emerging as novel antimicrobial agents, have exhibited exceptional potential in combating multidrug-resistant bacteria [36]. However, their recombinant expression in microbial systems is often hampered by their inherent toxicity to host cells, leading to limited titers. In this study, we explored the potential of RGG-mediated MLOs in mitigating the toxicity of AMPs against *C. glutamicum* (Fig. 4A). Two representative AMPs, melittin and lactoferricin B, were selected as model peptides [[37], [38], [39]]. For each protein, we designed constructs for both independent expression and N-terminal RGG fusion expression (Fig. S1).

Growth curve analysis revealed that cells independently expressing AMPs exhibited compromised growth compared to wild-type (WT) strains, whereas the RGG-fusion strain maintained comparable cell growth to the WT strains (Fig. 4B and D). This indicates that the observed growth defects were likely due to the cytotoxicity of AMPs rather than metabolic burden, as RGG-AMPs strains expressing both AMPs and the RGG domain, and therefore theoretically subject to a higher metabolic load, exhibited no significant growth defects. To further analyze protein expression, SDS-PAGE and Western blot (WB) were performed. In the non-phase separation (non-LLPS) group, AMPs expression was undetectable, likely due to the severe cytotoxicity of AMPs that limits their successful recombinant expression in microbial hosts. By contrast, RGG-fusion strains produced distinct protein bands at the expected molecular weights, confirming successful expression (Fig. 4C and E, Fig. S7A-S7D).

To further investigate whether LLPS-mediated MLOs formation contributes to this protective effect, we disrupted MLOs by adding 4 % (v/v) 1,6-hexanediol to the culture medium. Growth assays revealed that 1,6-hexanediol treatment impaired bacterial growth to varying extents, with a significantly more pronounced reduction observed in the RGG-fusion group compared to the non-fusion AMPs group (Fig. 4F). Specifically, RGG-fusion strains expressing melittin and lactoferricin B exhibited a 22.1 % and 25.4 % reduction in final OD_600_ values after hexanediol treatment, respectively, compared to only 9 % and 13.6 % reduction observed in non-fusion strains. This suggests that 1,6-hexanediol-induced disruption of MLOs may expose AMPs to the cytoplasmic environment, reinstating their toxic effects on host cells. SDS-PAGE analysis further supported this observation, as 1,6-hexanediol treatment significantly reduced AMPs expression levels (Fig. S7E, and Fig. S7F). Quantitative gray value analysis of protein bands revealed that melittin and lactoferricin B expression levels decreased by 54 % and 37 %, respectively, following 1,6-hexanediol treatment (Fig. 4G). These findings indicate that LLPS-mediated MLOs may help alleviate AMPs-induced cytotoxicity by physically sequestering AMPs and reducing their direct interactions with sensitive cellular components. When MLOs were disrupted, the toxic effects of AMPs were likely restored, leading to impaired growth and decreased protein expression.

Although the possibility that RGG functions as a general fusion tag to enhance protein expression cannot be entirely excluded, our findings suggest that RGG-mediated MLOs may play a potential role in reducing AMPs-induced cytotoxicity, thereby enabling their successful expression. One plausible mechanism is that phase-separated MLOs physically sequester AMPs, limiting their interactions with sensitive cellular components. This protective effect is supported by the observed disruption of MLOs upon 1,6-hexanediol treatment, which reinstated AMPs toxicity and led to reduced expression levels. The lack of detectable AMPs expression in the non-fusion strains may result from severe cytotoxicity that impairs cell viability or accelerates protein degradation, consistent with the observed growth defects in these strains. While the observed protective effect of RGG-mediated MLOs aligns with the proposed sequestration model, alternative explanations, such as improved peptide stability, transcription, or translation efficiency due to RGG fusion, remain viable and warrant further investigation.

## 3 Conclusion and discussion

LLPS-mediated MLOs have demonstrated great potential in regulating metabolic flux across various microbial systems such as *E. coli*, *B. subtilis*, and *Saccharomyces cerevisiae* [[6], [7], [8], [9]]. However, their application in *C. glutamicum* remains unexplored. In this study, we successfully engineered functional MLOs in *C. glutamicum* using the intrinsically disordered RGG domain from *C. elegans* LAF-1. While previous studies suggested that a single RGG domain alone might be insufficient to drive phase separation into condensates, our findings reveal that a single RGG domain is capable of mediating MLOs formation in *C. glutamicum*, aligning with similar observations in *E. coli* by Wan et al. [9]. This host-specific behavior of RGG domains likely reflects differences in cytoplasmic composition and molecular crowding among microbial chassis. Interestingly, increasing the copy number of RGG domains did not enhance MLOs formation efficiency, deviating from classical LLPS behavior. This divergence may stem from the unique cytoplasmic environment of *C. glutamicum* or the translational burden associated with expressing multiple RGG motifs.

To evaluate the metabolic engineering potential of these MLOs, we employed the indigoidine biosynthesis pathway as a proof-of-concept system. Indigoidine, synthesized via the condensation of two l-glutamine molecules, is particularly suited for production in *C. glutamicum* due to the host's native ability to overproduce l-glutamine [35]. Our results demonstrate that LLPS-mediated MLOs significantly enhanced indigoidine production in *C. glutamicum,* achieving titers 2.43-fold higher than the non-LLPS control. These findings highlight the potential of this approach for improving the synthesis of other metabolites. However, while the RGG-mediated MLOs played a key role in improving indigoidine titers, we cannot fully exclude the possibility that the RGG domain itself contributed to these effects via mechanisms beyond LLPS. Future studies incorporating control sequences that lack LLPS capacity, such as LLPS-deficient RGG mutants, will be essential to disentangle the specific contributions of LLPS and the intrinsic properties of the RGG domain.

To further optimize this system, several strategies could be explored. For instance, co-localizing bpsA with upstream enzymes involved in l-glutamine synthesis within MLOs could reduce intermediate diffusion losses and improve substrate channeling efficiency. This multi-enzyme co-localization strategy has been successfully implemented in other hosts like *S. cerevisiae* and *E. coli* and holds great promise for *C. glutamicum* [6,8]. Additionally, more flexible design strategies could be investigated, such as employing modular protein interaction tags (e.g., RIAD/RIDD or SpyCatcher/SpyTag) to minimize interference with target protein functionality, or incorporating light- or temperature-sensitive modules to dynamically regulate MLOs assembly and cargo protein release [6,8,16,40]. Although indigoidine biosynthesis represents a relatively simple single-enzyme pathway, the application of LLPS-mediated MLOs to more complex multi-enzyme networks remains a challenge. Such systems will require careful tuning of MLO assembly to avoid unintended effects on enzyme activity or pathway dynamics.

Beyond metabolic application, our results reveal that RGG-based condensates can mitigate the cytotoxicity of AMPs such as melittin and lactoferricin B. By sequestering toxic AMPs within RGG-based condensates, we successfully facilitated their recombinant expression. This toxin-shielding effect is further supported by the reduction in AMPs expression levels and host growth following treatment with 1,6-hexanediol, a chemical known to disrupt LLPS. These findings suggest that LLPS-mediated spatial isolation likely plays a key role in mitigating AMPs toxicity. However, other factors, such as changes in AMPs stability or folding introduced by RGG fusion, cannot be ruled out. To further investigate these mechanisms, future studies could utilize advanced imaging techniques or LLPS-disrupting RGG mutants to directly visualize AMPs sequestration within MLOs. Additionally, the role of RGG in altering AMPs activity or stability should be systematically evaluated.

In summary, we successfully constructed RGG-mediated MLOs in *C. glutamicum* and demonstrated their effectiveness in enhancing indigoidine biosynthesis. Furthermore, we pioneered the use of these MLOs to mitigate the cytotoxic effects associated with recombinant AMPs expression. These findings offer novel strategies and methodologies for metabolic engineering in *C. glutamicum* and showcase the expansive potential of LLPS in industrial biotechnology.

## 4 Materials and methods

### 4.1 Strains, plasmids, and cultural conditions

The bacterial strains and plasmids used in this study were listed in Table S1. Plasmid construction was performed in *E. coli* JM109. *C. glutamicum* CGMCC1.15647 was used as the host for protein expression. *E. coli* JM109 cells were cultured in Luria-Bertani (LB) medium or on LB plates containing 2 % (w/v) agar at 37 °C with shaking at 220 rpm. For *C. glutamicum* strains, the transformation process employed LBHIS medium, which consists of 5 g tryptone, 5 g NaCl, 2.5 g yeast extract, 91 g sorbitol, and 18.5 g brain heart infusion per liter, adjusted to pH 7.0. Unless otherwise specified, *C. glutamicum* strains were grown in LBB medium (LB broth supplemented with 10 g of brain heart infusion per liter) at 30 °C with shaking at 220 rpm. Chloramphenicol was added to the culture medium at a final concentration of 10 mg/L for *C. glutamicum* and 30 mg/L for *E. coli*. For inducible expression experiments, Isopropyl β-d-1-thiogalactopyranoside (IPTG) was added to the *C. glutamicum* cultures at a final concentration of 1 mM once the OD_600_ of the cells reached approximately 1.0.

For phase separation optimization, the experimental parameters were set as follows. For pre-induction time optimization, the pre-induction period was set to 2, 4, 6, and 8 h, with an IPTG concentration of 0.1 mM and an induction temperature of 30 °C. For inducer concentration optimization, the pre-induction time was fixed at 6 h, the induction temperature at 30 °C, and IPTG was added at final concentrations of 0.1, 0.2, 0.4, and 0.8 mM. For induction temperature optimization, temperatures were tested at 20 °C, 25 °C, 30 °C, and 35 °C. Pre-cultivation was conducted for 6 h at 30 °C, after which the temperature was shifted to the designated induction temperature. The final IPTG concentration was 0.2 mM.

### 4.2 Plasmid construction

All primers used in this study are listed in Table S2, and the plasmid P_btac_-HT11 served as the backbone for plasmid construction [27]. Codon-optimized DNA sequences for the RGG domain, bpsA, melittin, and lactoferricin B were synthesized by Sangon Biotech Co., Ltd. (Shanghai, China) and are provided in Table S3. To enable efficient detection of protein expression, a 6 × His tag was appended to the C-terminus of all expressed proteins. For the assembly of individual protein expression cassettes, the corresponding protein sequences were inserted into the linearized P_btac_-HT11 vector (*Hin*dIII/*Eco*RI) via homologous recombination. In the case of RGG fusion proteins, both the RGG domain and the individual protein sequences were simultaneously recombined into the same vector. Given that the RGG domain is an intrinsically disordered region, no linker was introduced between the proteins and the RGG domain in this study (Fig. S1) [16]. All DNA manipulations, including PCR amplification, restriction enzyme digestion, ligation, and Gibson assembly, were carried out according to standard protocols. The plasmids successfully constructed in *E. coli* JM109 were subsequently introduced into *C*. *glutamicum* CGMCC1.15647 through electroporation. The details of the electroporation procedure refer to the previous study by Ruan et al [41].

### 4.3 EGFP intensity measurement

Overnight cultures of *C. glutamicum* were inoculated at 2 % (v/v) into 10 mL of LBB medium and cultivated at 30 °C with shaking at 220 rpm until the OD_600_ of the cells reached approximately 1.0. IPTG was then added to a final concentration of 1 mM. After 30 h of induction cultivation under the same conditions, the OD_600_ values and fluorescence intensity of cells harboring EGFP-expressing plasmids were determined using a Synergy H4 microplate reader (BioTek, USA). The excitation and emission wavelengths for EGFP were 488 nm and 535 nm, respectively. All cultivation and measurement procedures were carried out in triplicate. The specific fluorescence intensity for each sample was calculated by normalizing the total fluorescence intensity to the corresponding OD_600_ value.

### 4.4 SDS-PAGE and western blot (WB) analysis

Cultivation conditions were the same as those described in the EGFP intensity measurement experiment. Cells were harvested via centrifugation at 12,000 *g* for 10 min at 4 °C. The cell pellets were then lysed through sonication on ice at 30 % output power, using pulse cycles of 1-s bursts followed by 2-s intervals to ensure efficient disruption while minimizing protein denaturation. The resulting lysates were clarified by centrifugation at 12,000 *g* for 15 min, and the supernatants were collected for further analysis. The 10 μL protein samples were analyzed using 12 % (w/v) sodium dodecyl sulfate-polyacrylamide gel electrophoresis (SDS-PAGE). The proteins separated by SDS-PAGE were then transferred onto a polyvinylidene fluoride (PVDF) membrane using a Trans-Blot apparatus (Bio-Rad, USA). The membrane was blocked by incubation with 5 % non-fat milk powder for 2 h to prevent non-specific binding. Subsequently, the blocking solution was replaced with a monoclonal horseradish peroxidase (HRP)-conjugated anti-His_6_ antibody. After a 1 h incubation, the membrane was washed three times with Tris-buffered saline with Tween 20 (TBST). Protein bands were visualized using an ECL detection kit (Amersham Biosciences, USA).

### 4.5 Relative protein expression level analysis

For the analysis of relative protein expression levels, bacterial cells with an OD_600_ of 10 were collected and disrupted by sonication. The supernatant was obtained by centrifugation and quantified using the BCA protein assay kit. Equal amounts of protein from each sample were separated by SDS-PAGE electrophoresis. The intensity of the target protein bands was analyzed using ImageJ software. For relative protein expression analysis, the protein expression level in the control group was set to 1. Relative protein expression levels in the experimental groups were calculated by normalizing the band intensity of each sample to that of the corresponding control.

### 4.6 Phase separation assay in vitro

The phase separation behavior was investigated using *C. glutamicum* expressing RGG-EGFP fusion protein. Bacterial cells were cultured under induction conditions and harvested by centrifugation at 4 °C, followed by resuspension in PBS buffer. The bacterial pellets were subjected to ultrasonication on ice, followed by centrifugation at 12,000 *g* for 20 min to collect the clarified supernatant. The His-tagged RGG-EGFP protein was purified via nickel-affinity chromatography (Ni-NTA resin). Protein concentration was quantified using a BCA assay kit. To evaluate phase separation dynamics, the purified proteins were diluted to various concentrations (1, 2, 4, 8, and 16 μM) in assay buffer supplemented with different NaCl concentrations (200, 400, and 600 mM). Reactions were incubated at 25 °C for 30 min, and LLPS-mediated MLOs was visualized by fluorescence microscopy.

### 4.7 Microscopy and image analysis

Fluorescence microscopy imaging of *C. glutamicum* cells was performed using the Zeiss LSM980 Laser Scanning Confocal Microscope with Two-photon Laser, SpinSR10 Spinning Disk Confocal Live Cell Imaging System, and EVOS FL Auto automated fluorescence imaging system. Sample preparation steps are as follows: Cells were collected by centrifugation at 10,000 *g* for 2 min, and the resulting pellet was fixed in Karnovsky's fixative (pH 7.3) for 10 min. The fixed cells were then centrifuged again and washed twice with 1 mL of PBS. Fixed cells were resuspended in 1 mL of PBS, and approximately 5 μL of the suspension was transferred onto a clean glass slide for imaging. EGFP fluorescence was excited using a 488 nm laser and a 561 nm excitation laser. Intracellular condensate analysis was performed using ImageJ software.

### 4.8 Fluorescence recovery after photobleaching (FRAP) analysis

FRAP experiments were performed using a Zeiss LSM 980 confocal microscope. Samples were loaded onto glass slides and excited at 488 nm with 2 % laser power to achieve appropriate green fluorescence intensity. The center of the droplet was then photobleached using an 8 ms laser pulse at 100 % laser power for approximately 1 s. Post-bleaching recovery was monitored by imaging at 25 °C with a frame rate of 0.5 s per frame for 3 min to record fluorescence recovery. Fluorescence intensities were measured within the bleached region of interest (ROI 1) and a control region (ROI 2) that was not photobleached. To analyze recovery, the fluorescence intensity in ROI 1 was compared to the average intensity in the same region before bleaching. Specifically, the average fluorescence intensity in ROI 1 from the frames recorded immediately before bleaching was set as the baseline value (defined as 1). The fluorescence recovery after bleaching was then expressed as a relative change compared to this baseline. A control region (ROI 2) was used to ensure that any changes in fluorescence were not caused by imaging conditions or other external factors.

### 4.9 Urea and 1,6-hexanediol treatment

For the urea perturbation assays, 1 mL of cell culture was collected by centrifugation at 10,000 *g* and washed twice with 50 mM PBS buffer (pH 7.4). Then, the cell pellets were resuspended in 50 mM PBS, 50 mM PBS with 0.5 M urea, and 50 mM PBS with 1 M urea, respectively. The suspensions were incubated at 25 °C for 20 min. Following incubation, the cells were washed twice with 50 mM PBS and subsequently prepared for microscopy observation. For 1,6-hexanediol treatment, after culturing at 30 °C for 6 h, the culture was treated with 0.2 mM IPTG and different concentrations of 1,6-hexanediol (2 %, 3 %, 4 %, and 5 % v/v), and then incubated at 25 °C for 30 h before sampling for analysis.

### 4.10 Measurement of indigoidine production

The absorption spectrum of indigoidine was determined by performing a full-wavelength scan (500–750 nm) to identify its characteristic absorption peak. The optimal wavelength identified was used for subsequent quantitative measurements. Sample processing, measurement, and standard curve preparation were carried out following the method previously described by Ghiffary et al. [35].

## CRediT authorship contribution statement

**Manman Sun:** Writing – review & editing, Writing – original draft, Visualization, Supervision, Methodology, Data curation, Conceptualization. **Yimeng Zhao:** Validation, Software. **Rodrigo Ledesma-Amaro:** Writing – review & editing, Supervision, Resources. **Jin Gao:** Formal analysis, Software, Writing – review & editing. **Xiuxia Liu:** Writing – review & editing, Formal analysis. **Zhonghu Bai:** Writing – review & editing, Resources, Investigation. **Alex Xiong Gao:** Writing – review & editing, Supervision, Methodology, Funding acquisition, Conceptualization. **Peng Wang:** Writing – review & editing, Supervision, Resources, Funding acquisition.

## Ethical approval

This article does not contain any study with human participants or animals by any of the authors.

## Declaration of competing interest

The authors declare that they have no known competing financial interests or personal relationships that could have appeared to influence the work reported in this paper.

## References

[1] Scown C.D., Keasling J.D. (2022). Sustainable manufacturing with synthetic biology. Nat Biotechnol.

[2] Mao J., Zhang H., Chen Y., Wei L., Liu J., Nielsen J. (2024). Relieving metabolic burden to improve robustness and bioproduction by industrial microorganisms. Biotechnol Adv.

[3] Cravens A., Payne J., Smolke C.D. (2019). Synthetic biology strategies for microbial biosynthesis of plant natural products. Nat Commun.

[4] Sun M., Gao A.X., Ledesma-Amaro R., Li A., Wang R., Nie J. (2022). Hypersecretion of OmlA antigen in *Corynebacterium glutamicum* through high-throughput based development process. Appl Microbiol Biotechnol.

[5] Duan Y., Zhai W., Liu W., Zhang X., Shi J.-S., Zhang X. (2021). Fine-tuning multi-gene clusters via well-characterized gene expression regulatory elements: case study of the arginine synthesis pathway in *C. glutamicum*. ACS Synth Biol.

[6] Zhao E.M., Suek N., Wilson M.Z., Dine E., Pannucci N.L., Gitai Z. (2019). Light-based control of metabolic flux through assembly of synthetic organelles. Nat Chem Biol.

[7] Yu W., Jin K., Wang D., Wang N., Li Y., Liu Y. (2024). De novo engineering of programmable and multi-functional biomolecular condensates for controlled biosynthesis. Nat Commun.

[8] Wang Y., Liu M., Wei Q., Wu W., He Y., Gao J. (2022). Phase-separated multienzyme compartmentalization for terpene biosynthesis in a prokaryote. Angew Chem Int Ed.

[9] Wan L., Zhu Y., Ke J., Zhang W., Mu W. (2024). Compartmentalization of pathway sequential enzymes into synthetic protein compartments for metabolic flux optimization in *Escherichia coli*. Metab Eng.

[10] Dzuricky M., Rogers B.A., Shahid A., Cremer P.S., Chilkoti A. (2020). De novo engineering of intracellular condensates using artificial disordered proteins. Nat Chem.

[11] Gabryelczyk B., Alag R., Philips M., Low K., Venkatraman A., Kannaian B. (2022). In vivo liquid–liquid phase separation protects amyloidogenic and aggregation-prone peptides during overexpression in *Escherichia coli*. Protein Sci.

[12] Garabedian M.V., Wang W., Dabdoub J.B., Tong M., Caldwell R.M., Benman W. (2021). Designer membraneless organelles sequester native factors for control of cell behavior. Nat Chem Biol.

[13] Ma S., Liao K., Li M., Wang X., Lv J., Zhang X. (2023). Phase-separated DropCRISPRa platform for efficient gene activation in Mammalian cells and mice. Nucleic Acids Res.

[14] Alberti S., Gladfelter A., Mittag T. (2019). Considerations and challenges in studying liquid-liquid phase separation and biomolecular condensates. Cell.

[15] Zhang H., Ji X., Li P., Liu C., Lou J., Wang Z. (2020). Liquid-liquid phase separation in biology: mechanisms, physiological functions and human diseases. Sci China Life Sci.

[16] Wan L., Zhu Y., Zhang W., Mu W. (2023). Phase-separated synthetic organelles based on intrinsically disordered protein domain for metabolic pathway assembly in *Escherichia coli*. ACS Nano.

[17] Gabryelczyk B., Sammalisto F.E., Gandier J.A., Feng J., Beaune G., Timonen J.V.I. (2022). Recombinant protein condensation inside *E. coli* enables the development of building blocks for bioinspired materials engineering – biomimetic spider silk protein as a case study. Mater Today Bio.

[18] Elbaum-Garfinkle S., Kim Y., Szczepaniak K., Chen C.C.H., Eckmann C.R., Myong S. (2015). The disordered P granule protein LAF-1 drives phase separation into droplets with tunable viscosity and dynamics. Proc Natl Acad Sci USA.

[19] Freudl R. (2017). Beyond amino acids: use of the *Corynebacterium glutamicum* cell factory for the secretion of heterologous proteins. J Biotechnol.

[20] Liu X.X., Li Y., Bai Z.H. (2021). Microbial cell factories engineering for production of biomolecules.

[21] Liu X., Zhang W., Zhao Z., Dai X., Yang Y., Bai Z. (2017). Protein secretion in *Corynebacterium glutamicum*. Crit Rev Biotechnol.

[22] Liu J., Liu M., Shi T., Sun G., Gao N., Zhao X. (2022). CRISPR-Assisted rational flux-tuning and arrayed CRISPRi screening of an L-proline exporter for L-proline hyperproduction. Nat Commun.

[23] Wang Y., Cheng H., Liu Y., Liu Y., Wen X., Zhang K. (2021). In-situ generation of large numbers of genetic combinations for metabolic reprogramming via CRISPR-Guided base editing. Nat Commun.

[24] Liu J., Zhao X., Cheng H., Guo Y., Ni X., Wang L. (2025). Comprehensive screening of industrially relevant components at genome scale using a high-quality gene overexpression collection of *Corynebacterium glutamicum*. Trends Biotechnol.

[25] Yu X., Li S., Feng H., Liao X., Xing X.H., Bai Z. (2023). CRISPRi-microfluidics screening enables genome-scale target identification for high-titer protein production and secretion. Metab Eng.

[26] Liu X., Sun M., Gao A.X., Ledesma-Amaro R., Fang Q., Yang Y. (2023). Leaderless bicistronic design for precise and reliable control of gene expression in *Corynebacterium glutamicum*. ACS Synth Biol.

[27] Sun M., Gao X., Zhao Z., Li A., Wang Y., Yang Y. (2020). Enhanced production of recombinant proteins in *Corynebacterium glutamicum* by constructing a bicistronic gene expression system. Microb Cell Fact.

[28] Elowitz M.B., Levine A.J., Siggia E.D., Swain P.S. (2002). Stochastic gene expression in a single cell. Science.

[29] Taniguchi Y., Choi P.J., Li G.W., Chen H., Babu M., Hearn J. (2010). Quantifying *E. coli* proteome and transcriptome with single-molecule sensitivity in single cells. Science.

[30] Ozbudak E.M., Thattai M., Kurtser I., Grossman A.D., Van Oudenaarden A. (2002). Regulation of noise in the expression of a single gene. Nat Genet.

[31] Schuster B.S., Reed E.H., Parthasarathy R., Jahnke C.N., Caldwell R.M., Bermudez J.G. (2018). Controllable protein phase separation and modular recruitment to form responsive membraneless organelles. Nat Commun.

[32] Wei S.P., Qian Z.G., Hu C.F., Pan F., Chen M.T., Lee S.Y. (2020). Formation and functionalization of membraneless compartments in *Escherichia coli*. Nat Chem Biol.

[33] Li S., Wang Y., Lai L. (2023). Small molecules in regulating protein phase separation. Acta Biochim Biophys Sin.

[34] Düster R., Kaltheuner I.H., Schmitz M., Geyer M. (2021). 1,6-Hexanediol, commonly used to dissolve liquid-liquid phase separated condensates, directly impairs kinase and phosphatase activities. J Biol Chem.

[35] Ghiffary M.R., Prabowo C.P.S., Sharma K., Yan Y., Lee S.Y., Kim H.U. (2021). High-level production of the natural blue pigment indigoidine from metabolically engineered *Corynebacterium glutamicum* for sustainable fabric dyes. ACS Sustainable Chem Eng.

[36] Zheng S., Tu Y., Li B., Qu G., Li A., Peng X. (2025). Antimicrobial peptide biological activity, delivery systems and clinical translation status and challenges. J Transl Med.

[37] Chen Q.C., Liu L., Yu T.Y., Tang L., Yin M.L., Zhu W.H. (2021). High-level expression and purification of melittin in *Escherichia coli* using SUMO fusion partner. Int J Pept Res Therapeut.

[38] Huang H.Y., Hsu H.Y., Kuo C.Y., Wu M.L., Lai C.C., Chang G.R.L. (2024). Heterologous expressing melittin in a probiotic yeast to evaluate its function for promoting NSC-34 regeneration. Appl Microbiol Biotechnol.

[39] Lee B.C., Tsai J.C., Hung C.W., Lin C.Y., Sheu J.C., Tsai H.J. (2022). High antimicrobial activity of lactoferricin-expressing *Bacillus subtilis* strains. Microb Biotechnol.

[40] Li M., Park B.M., Dai X., Xu Y., Huang J., Sun F. (2022). Controlling synthetic membraneless organelles by a red-light-dependent singlet oxygen-generating protein. Nat Commun.

[41] Ruan Y., Zhu L., Li Q. (2015). Improving the electro-transformation efficiency of *Corynebacterium glutamicum* by weakening its cell wall and increasing the cytoplasmic membrane fluidity. Biotechnol Lett.

