# Supplementary material for: Engineered membraneless organelles in Corynebacterium glutamicum for enhanced indigoidine biosynthesis and antimicrobial peptide production
Source: Synth Syst Biotechnol. 2025 Aug 5;10(4):1331–40. doi: 10.1016/j.synbio.2025.08.001 (PMC12395988; doi:10.1016/j.synbio.2025.08.001)
Supplement: Multimedia component 1 [file mmc1.docx]

**Engineered Membraneless Organelles in *Corynebacterium glutamicum* for Enhanced Indigoidine Biosynthesis and Antimicrobial Peptide Production**

Manman Sun^1,2#^, Yimeng Zhao^3#^, Rodrigo Ledesma-Amaro^4^, Jin Gao^5^, Xiuxia Liu^6^, Zhonghu Bai^6^, Alex Xiong Gao^7*^, Peng Wang^1,2*^

^#^ These authors contributed equally to this work.

* Correspondence: [gaoxiong@ust.hk;](mailto:gaoxiong@ust.hk;) pengwang@ipp.ac.cn

^1^ Key laboratory of high magnetic field and Ion beam physical biology, Hefei Institutes of Physical Science, Chinese Academy of Sciences, Hefei 230031, China

^2^ Institute of Hefei Artificial Intelligence Breeding Accelerator, Hefei 230000, China

^3^ School of Food and Nutrition, Anhui Agricultural University, Hefei 230036, China

^4^ Department of Bioengineering and Imperial College Centre for Synthetic Biology, Imperial College London, London SW7 2AZ, UK

^5^ Department of Neurobiology and Cellular Biology, Xuzhou Medical University, Xuzhou 221004, Jiangsu, China

^6^ National Engineering Research Center of Cereal Fermentation and Food Biomanufacturing, Jiangnan University, Wuxi 214112, China

^7^ Division of Life Science, The Hong Kong University of Science and Technology, Hong Kong 999077, China


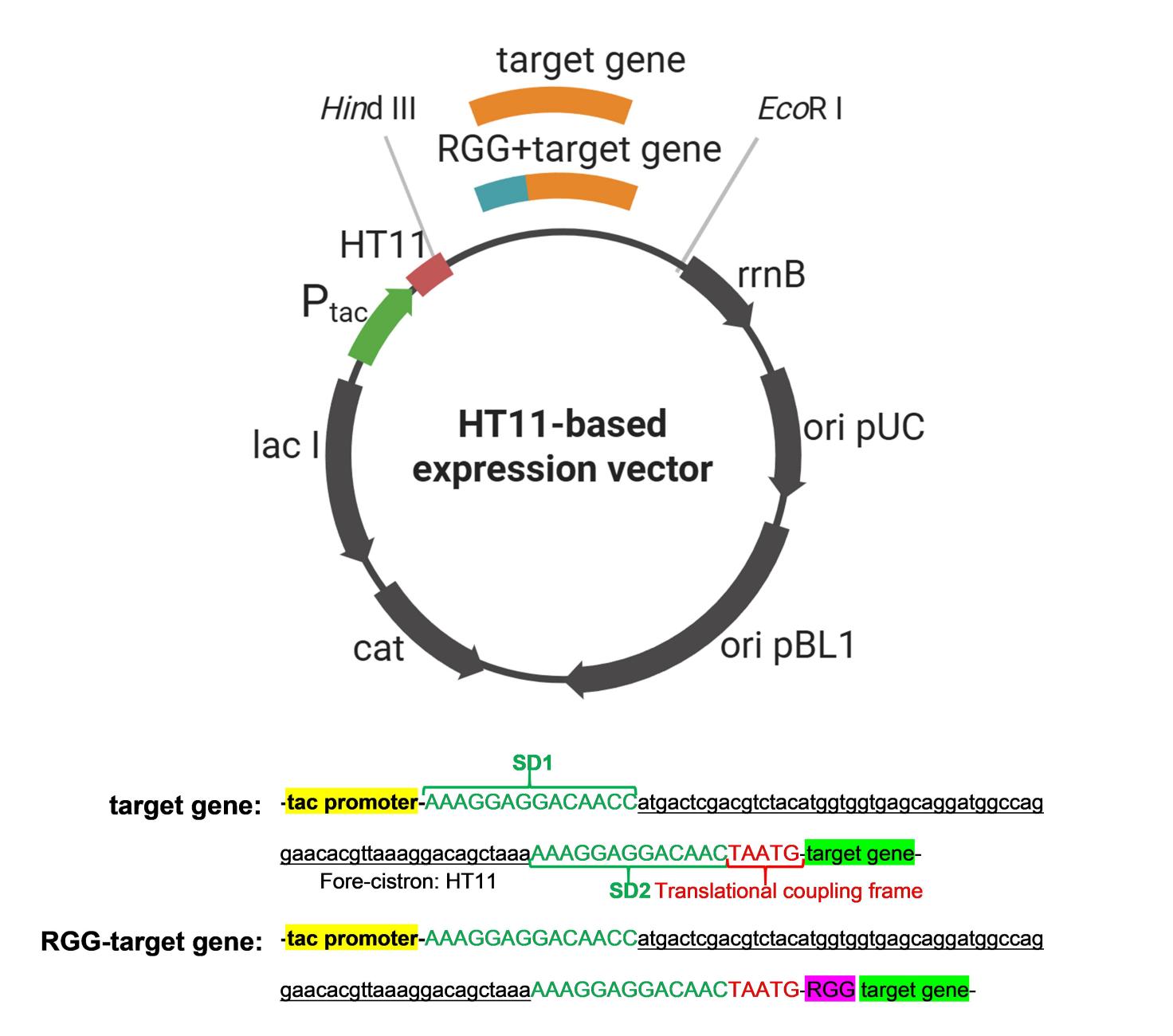


**Figure. S1 Schematic diagram of the expression vectors constructed in this study.**Note: All expression vectors were constructed based on the bicistronic expression vector Pbtac-HT11 developed in our previous study (Sun et al. 2020). HT11 is a coding sequence for a short peptide that is easy to translate and is inserted upstream of the target gene to enhance the expression level of the downstream gene through translational coupling. Since the RGG domain is an intrinsically disordered region, no linker was introduced between the proteins and the RGG domain in this study.


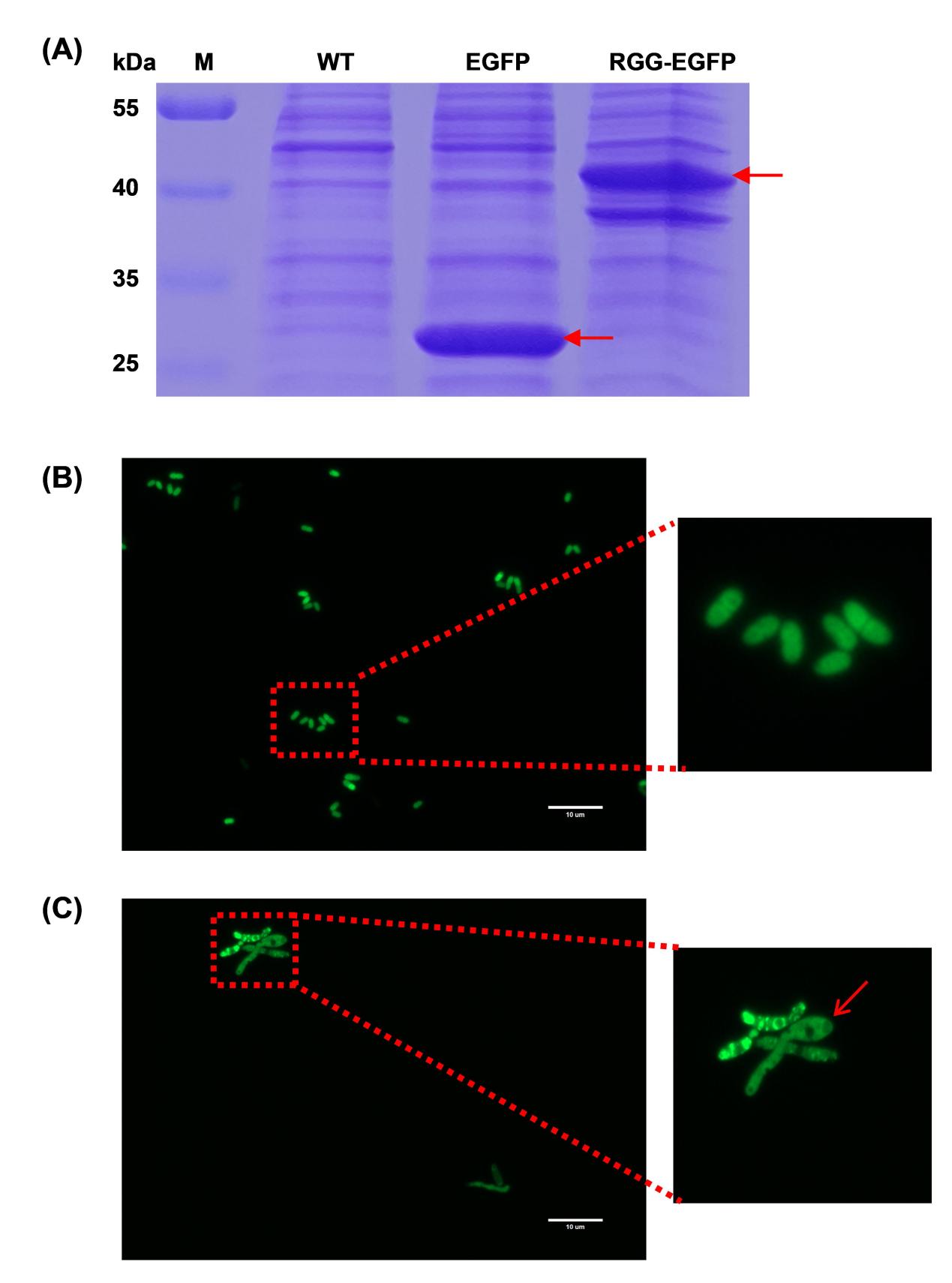


**Figure. S2 The effect of RGG-mediated membraneless organelles (MLOs) on the cell morphology of *C. glutamicum*.** (A) SDS-PAGE analysis of RGG-EGFP expression, (B) Microscopic observation of strains expressing EGFP, (C) Microscopic observation of strains expressing RGG-EGFP with enlarged and distorted morphology.


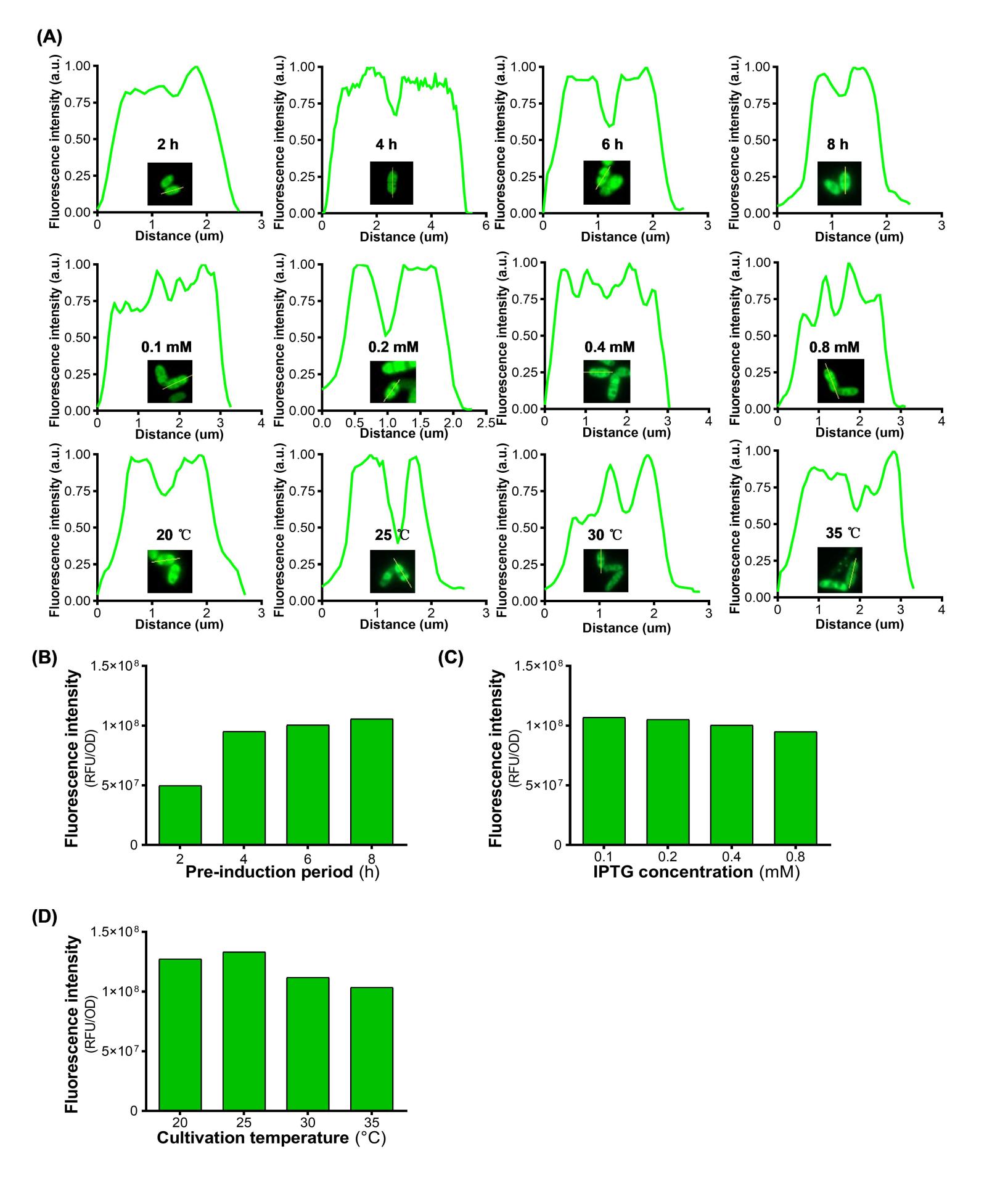


**Figure. S3 Optimization of cultivation conditions and characterization analysis of RGG-mediated MLOs.** (A) Fluorescence distribution analysis of strains expressing RGG-EGFP under different culture conditions. (B) Fluorescence intensity analysis of RGG-EGFP expressing strain under different pre-induction periods. (C) Fluorescence intensity analysis of RGG-EGFP expressing strain under different isopropyl β-D-1-thiogalactopyranoside (IPTG) concentrations. (D) Fluorescence intensity analysis of RGG-EGFP expressing strain under different cultivation temperatures.

**
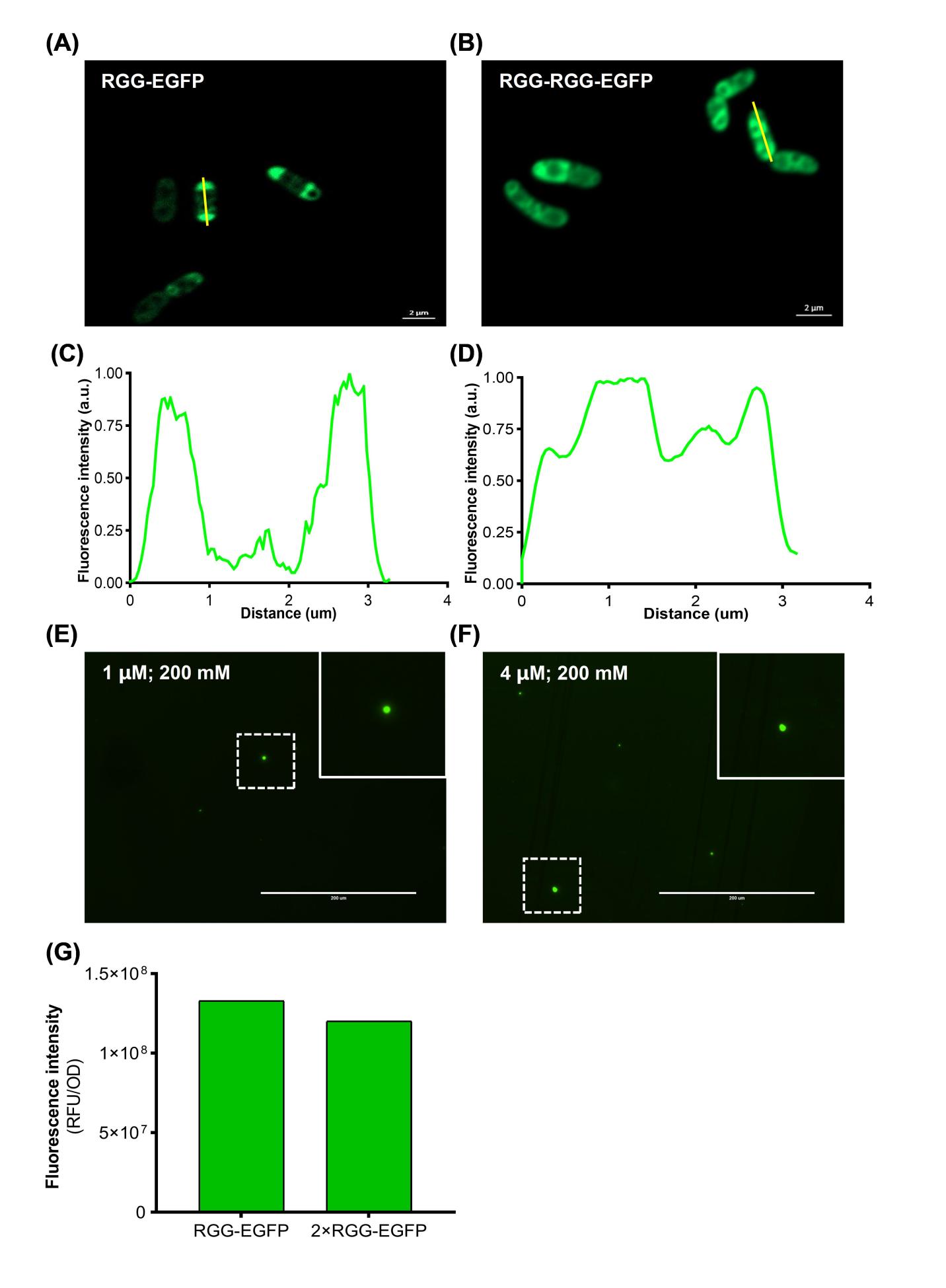
**

**Figure. S4 Microscopic observation of liquid-liquid phase separation (LLPS)-driven droplets in vivo and in vitro.** (A) Microscopic observation of strains expressing RGG-EGFP. (B) Microscopic observation of strains expressing RGG-RGG-EGFP. (C) Fluorescence distribution analysis of the RGG-EGFP expressing strain using ImageJ software. (D) Fluorescence distribution analysis of the RGG-RGG-EGFP expressing strain. (E) Formation of condensates by RGG-EGFP protein at 1 μM in vitro (200 mM NaCl). (F) Formation of condensates by RGG-EGFP protein at 4 μM in vitro (200 mM NaCl). (G) Fluorescence intensity analysis of strains expressing RGG-EGFP and RGG-RGG-EGFP.

**
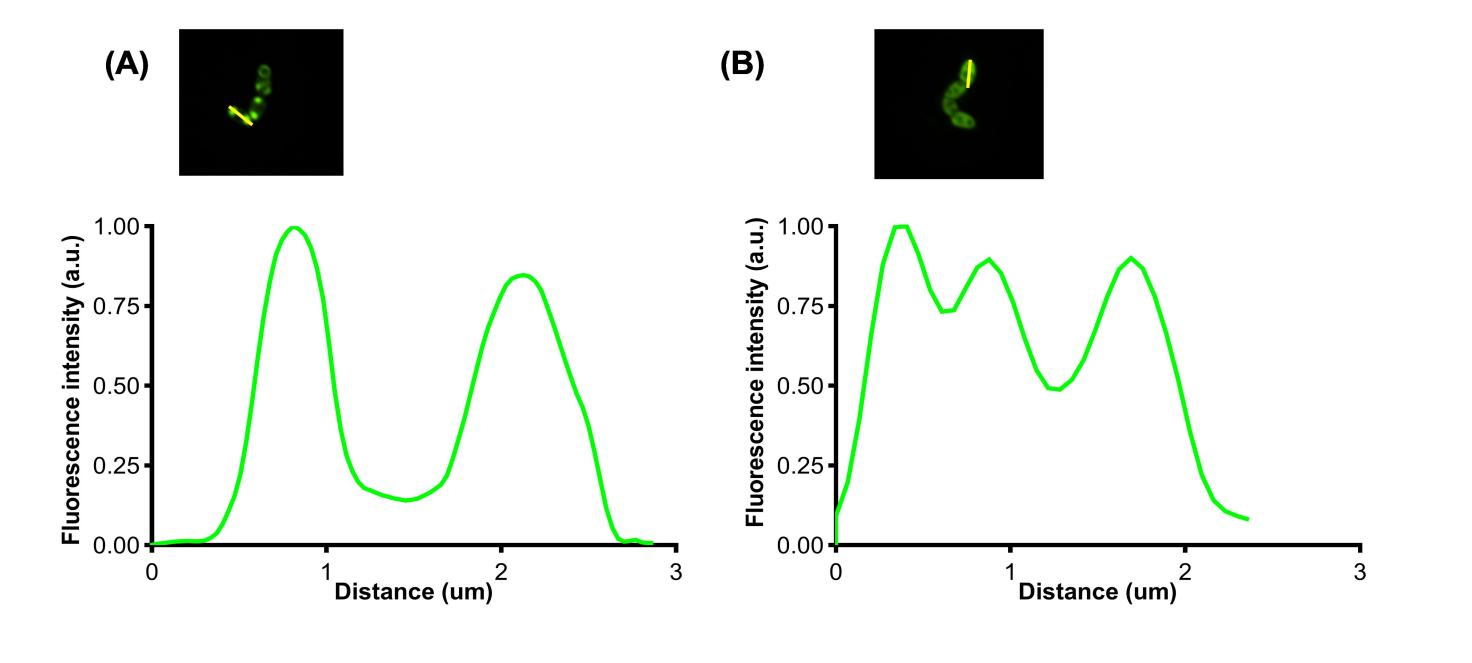
**

**Figure. S5 Fluorescence distribution analysis.** (A) Fluorescence distribution analysis of strains expressing the RGG-EGFP using ImageJ software. (B) Fluorescence distribution analysis of strains expressing the RGG-EGFP after 1,6-hexanediol treatment.


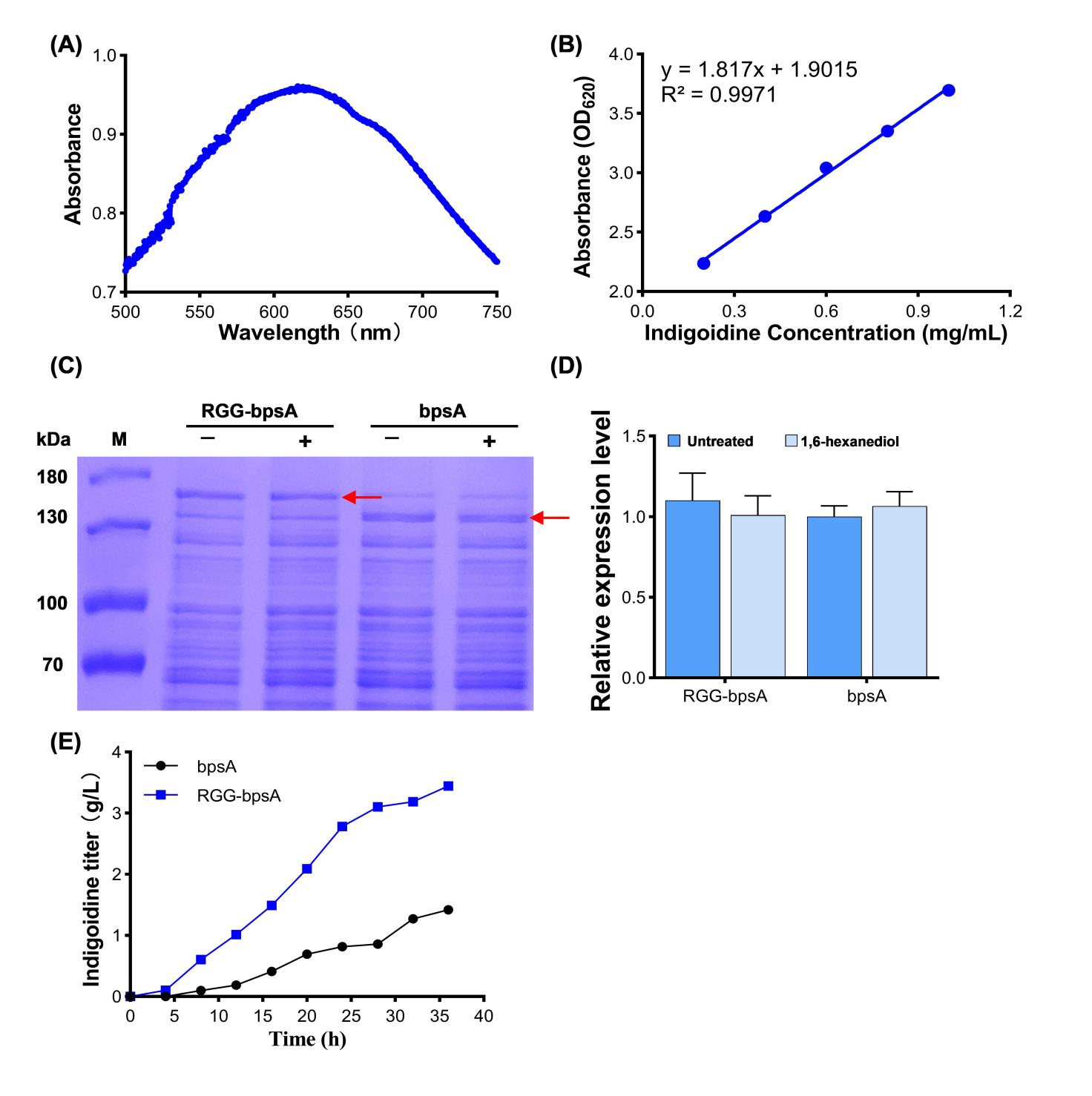


**Figure. S6 Impact of RGG fusion and 1,6-hexanediol treatment on indigoidine production and protein expression.** (A) Absorbance spectrum of indigodine measured across wavelengths from 500 nm to 750 nm. (B) Standard curve of indigodine concentration (mg/mL) versus absorbance at 620 nm. (C) Effect of RGG fusion and 1,6-hexanediol treatment on gene expression levels. Line M represents Marker. “+ ” represents 1,6-hexanediol treatment. (D) Quantitative analysis of band intensities from the SDS-PAGE gel shown in (C), with the expression level of untreated bpsA as the control, defined as 1. (E) Indigoidine titer at different time points for RGG-bpsA and bpsA strains.


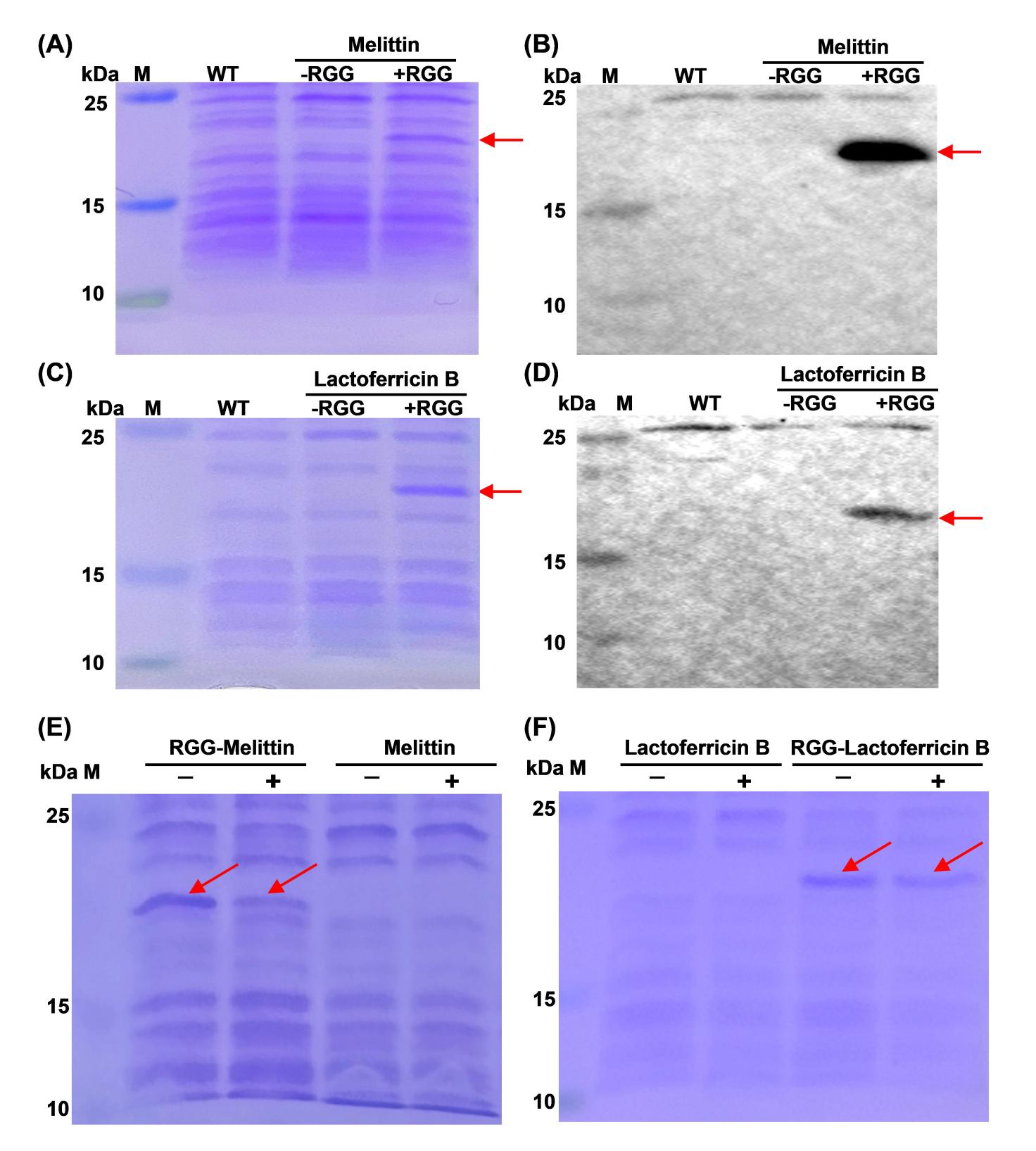


**Figure. S7 The effect of RGG fusion and 1,6-hexanediol treatment on AMPs expression.** (A), (B) SDS-PAGE and WB analysis of RGG-Melittin expression. (C), (D) SDS-PAGE and WB analysis of RGG-Lactoferricin B expression. (E),(F) Effect of 1,6-hexanediol treatment on AMPs expression. “+ ” represents 1,6-hexanediol treatment.

**Table S1** Bacteria strains and plasmids used in this study.

| **Strains or plasmids** | **Description** | **Sources** |
| --- | --- | --- |
| **Strains** |  |  |
| *E. coli* JM109 | K12-derived strain with recA1 and endA1 mutations; high transformation efficiency, suitable for plasmid construction molecular cloning, and plasmid amplification | Laboratory stock |
| *C. glutamicum* CGMCC1.15647 | Wild-type strain, Gram-positive, non-pathogenic, suitable host for recombinant protein expression | (Sun et al. 2022) |
| **Plasmids** |  |  |
| P_btac_-HT11 | Constructed based on the pXMJ19 *C. glutamicum*/*E. coli* shuttle vector, with a short peptide coding sequence inserted upstream of the multiple cloning site to create a bicistronic tac expression system, Chloramphenicol resistance, replication origins: ori pUC and ori pBL1, rrnB transcription terminator | (Sun et al. 2020) |
| P_btac_-HT11-EGFP | Containing a bicistronic tac promoter and the EGFP gene | (Sun et al. 2020) |
| P_btac_-HT11-RGG | Containing a bicistronic tac promoter and the RGG gene | This study |
| P_btac_-HT11-RGG-EGFP | Containing a bicistronic tac promoter and expressing the RGG-EGFP fusion protein | This study |
| P_btac_-HT11-RGG-RGG-EGFP | Containing a bicistronic tac promoter and expressing the RGG-RGG-EGFP fusion protein | This study |
| P_btac_-HT11-bpsA | Containing a bicistronic tac promoter and expressing the bpsA protein | This study |
| P_btac_-HT11-RGG-bpsA | Containing a bicistronic tac promoter and expressing the RGG-bpsA fusion protein | This study |
| P_btac_-HT11-Melittin | Containing a bicistronic tac promoter and expressing the Melittin protein | This study |
| P_btac_-HT11-RGG-Melittin | Containing a bicistronic tac promoter and expressing the RGG-Melittin fusion protein | This study |
| P_btac_-HT11-Lactoferricin B | Containing a bicistronic tac promoter and expressing the Lactoferricin B protein | This study |
| P_btac_-HT11--RGG-Lactoferricin B | Containing a bicistronic tac promoter and expressing the RGG-Lactoferricin B fusion protein | This study |

**Table S2 Primers used in this study**

| **Primer** | **Sequence (5′-3′)^a^** | **Intension** |
| --- | --- | --- |
| RGG-F | AAGGACAGCTAAAAAAGGAGGACAACTAATGATGCCACGCGAATCCAACCAGTC | Amplifying RGG for P_btac_-HT11-RGG-EGFP |
| RGG-R | GCTCCTCGCCCTTGCTCACCATAAGCTTACCGCCGTTATCGCGGCGATCG | Amplifying RGG for P_btac_-HT11-RGG-EGFP |
| EGFP-F | GATCGCCGCGATAACGGCGGTAAGCTTATGGTGAGCAAGGGCGAGGAG | Amplifying EGFP |
| EGFP-R | CATCCGCCAAAACAGCCAAGCTGAATTCTTACTTGTACAGCTCGTCCATG | Amplifying EGFP |
| RGG-RGG-F | TCGCCGCGATAACGGCGGTATGCCACGCGAATCCA | Amplifying RGG for P_btac_-HT11-RGG-EGFP |
| RGG-RGG-R | CCCTTGCTCACCATAAGCTTACCGCCGTTATCGCGGCGATCG | Amplifying RGG for P_btac_-HT11-RGG-EGFP |
| bpsA-F | AAAGGAGGACAACTAATGAAGCTTATGACCCTGCAAGAAACCTCT | Amplifying bpsA for independent expression |
| RGG-bpsA-F | ATCGCCGCGATAACGGCGGTAAGCTTATGACCCTGCAAGAAACCTCTGTGCTGGAA | Amplifying bpsA for RGG-bpsA fusion expression |
| bpsA-R | CAAAACAGCCAAGCTGAATTCTCATTCGCCCAGCAGGTAGCGGAT | Amplifying bpsA |
| Melittin-F | GGAGGACAACTAATGAAGCTTATGGGCATCGGTGCCGTGCTTAAA | Amplifying Melittin for independent expression |
| RGG-Melittin-F | ATCGCCGCGATAACGGCGGTAAGCTTATGGGCATCGGTGCCGTGCTTAAA | Amplifying Melittin for RGG-bpsA fusion expression |
| Melittin-R | CCAAAACAGCCAAGCTGAATTCTTAATGGTGATGGTGATGG | Amplifying Melittin |
| Lactoferricin B-F | AAAGGAGGACAACTAATGAAGCTTATGTTCAAATGTCGGCGCTGGCAGTGGCGC | Amplifying Lactoferricin B for independent expression |
| RGG-Lactoferricin B-F | AACGATCGCCGCGATAACGGCGGTAAGCTTATGTTCAAATGTCGGCGCTGGCAGTGGCGC | Amplifying Lactoferricin B for RGG-Lactoferricin B fusion expression |
| Lactoferricin B-R | CAAAACAGCCAAGCTGAATTCTTAATGGTGATGGTGATGGTGAAAGGCTCGACGCACGCA | Amplifying Lactoferricin B |
| P19-seq-F | TCTCCGGGAGCTGCATGTGTCAG | Upstream sequencing primer |
| P19-seq-R | CAGGAGAGCGTTCACCGACAAACA | Downstream sequencing primer |

**^a^** The red bases represent homologous arms

**Table S3 Codon-optimized DNA sequences used in this study**

| **Protein** | **Sequence (5′-3′)^a^** |
| --- | --- |
| RGG | ATGCCACGCGAATCCAACCAGTCCAACAACGGCGGTTCCGGTAACGCAGCCTTGAACCGTGGTGGTCGCTACGTTCCACCACACTTGCGCGGTGGTGATGGCGGTGCAGCAGCAGCTGCATCCGCAGGCGGTGATGATCGTCGAGGTGGTGCAGGTGGTGGCGGCTACCGCCGAGGTGGCGGTAACTCCGGTGGAGGCGGAGGTGGTGGTTACGATCGCGGCTATAACGATAACCGCGATGATCGCGATAACCGAGGTGGTTCCGGTGGCTACGGTCGCGATCGCAACTACGAAGACCGCGGATACAACGGTGGCGGCGGTGGTGGTGGCAACCGCGGCTACAACAACAACCGCGGTGGCGGTGGAGGTGGCTACAACCGCCAAGATCGCGGTGATGGTGGCTCTTCCAACTTCTCTCGCGGCGGTTACAACAATCGCGATGAAGGCTCCGATAACCGTGGCTCCGGTCGCTCTTACAACAACGATCGCCGCGATAACGGCGGTTAA |
| bpsA | ATGACCCTGCAAGAAACCTCTGTGCTGGAACCAACCCTGCGCGGCACCACTACCCTGCCTGATCTGCTGGCAAAACGCGTGGCAGAACACCCTGAAGCAACCGCAGTGGCATACCGCGATGAAAAGCTGACCTACCGCGAGCTGGCATCCCGCTCCTCCGCACTGGCAGAATACCTGCGCCACCTGGGTGTGTCCACCGATGATTGCGTGGGCCTGTTCGTCGAACCATCCATCGATCTGATGGTGGGCGCATGGGGCATCCTGTCCGCCGGCGCAGCATACCTGCCTCTGTCTCCTGAATACCCTGAAGATCGCCTGCGCTACATGATCGAGAACTCCCAAGCAAAGATCATCCTGGCACAGCAGCGCCTGGTGACCCGCCTGCGCGAACTGGCACCTCAAGATGTGCGCGTGGTGACCCTGCGCGAATCCGAAGCATTCGTGCTGCCTGAAGGCCAAGTGGCACCTGCAATCGAAGGCGCACGCCCTGATTCCCTGGCATACGTGATCTACACCTCCGGCTCCACCGGCAAGCCAAAGGGCGTGATGATCGAACACCACTCCATCGTGTCTCAGCTGGGCTGGCTGCGCGAAACCTACGGTATCGATCGCTCCAAGACCATCCTGCAAAAGACCCCAATGTCCTTCGATGCAGCACAGTGGGAAATCCTGTCCCCTGCAAACGGCGCAACCGTGGTGATGGGCGCACCTGGCGTGTACGCAGATCCTGAAGGCCTGATCGAAACCATCGTGAAGTACGGCGTGACCACCCTGCAATGCGTGCCAACCCTGCTGCAAGGCCTGCTGGATACCGAAAAGTTCCCTGAATGCACCTCCCTGCAACAGATCTTCTCCGGCGGCGAAGCACTGTCCCGCCTGCTGGCAATTCAGACCACCCAAGAAATGCCTGGCCGCGCACTGATCAACGTGTACGGCCCAACCGAATGCACCATCAACTCCTCTTCCTACGCAGTCGATCCTGCCGAACTGGGCGAAGCACCACAGTCCATCTCCATCGGCGCACCTGTGGCAGATACCGAATACCACATCCTGGGCAAGGAAGATCTGAAGCCTGTGGGCGTGGGCGAGATCGGCGAACTCTACATCGGCGGTGGTCAGCTGGCACGTGGTTACCTGCACCGCCCTGATCTGACCGCAGAACGCTTCCTGGAGATCGAAGTGACCGAAGGCGCCGGCCCTGTGCGCCTGTACAAGACCGGCGATCTGGGTCAGTGGAACCCTGATGGCACCGTGCAGTTCGCCGGCCGCGCAGATAACCAAGTGAAGCTGCGCGGCTACCGCGTGGAACTGGATGAAATCTCCCTGGCAATCGAAAACCACGATTGGGTGCGCAACGCAGCAGTGATCGTGAAGAACGATGGCCGCACCGGCTTTCAGAACCTGATCGCATGCGTGGAACTGTCCGAAAAGGAAGCAGCACTGATGGATCAAGGCAACCACGGCTCCCACCACGCATCCAAGAAGTCCAAGCTGCAAGTGAAGGCACAGCTGTCCAACCCTGGCCTGCGCGATGATGCCGATCTGGCCGCCCGCGTGGCATATGATCTGCCTGGCGCAGAACCAACCCCTGAACAGCGCTCCCGCGTGTTCGCACGCAAGACCTACCGCTTCTACGAAGGCGGCGCAGTGACCGAAGCAGACCTGCTGGCACTGCTGGGCGGCCAAGTGCCAGCAGCATACTCCCGCAAGGCAGCAGATCTGGCACCAGCAGAACTCGGTCAGATCCTGCGCTGGTTCGGTCAGTACCTGTCCGAAGAACGCCTGCTGCCAAAGTACGGCTACGCATCCCCTGGCGCACTGTACGCAACTCAGCTGTACTTCGAACTGGAAGGCGTGGGCGGCCTGCAACCTGGCTACTATTACTATCAGCCACAGCGCCATCAGCTGGTGCTGATCTCCGAAAAGGCAGCAACCGGCCGCCCAACCGCACACATCCACTTCATCGGCAAGCGCGGCGGCATCGAACCTGTGTACAAGAACAACATCCAAGAAGTGCTGGAAATCGAAACCGGCCACATCGTCGGCCTGTTCGAACAAGTGCTGCCTGCATACGGCCTGGACATCCGCGATCTGGCATACGAACCAGCAGTGCGCGATCTGCTGGATGTGCCTGAAGAAGATTTCTACCTGGGCACCTTCGAACTGGTGCCACACACCGGCCGCCGCGAAGATCACGCAGAAGTGTACGTGCAGACCCACGGCTCCAAGGTGGCAAACCTGCCTGAAGGTCAGTACCGCTACGCAGATGGCACCCTGACCCGCTTCTCCGATGACATCGTGCTGAAGAAGCAAGTGATCGCAATCAATCAGTCCGTGTACCAAGCAGCATCCTTCGGCATCTCCGTGATCTCCCGCGCACCTGAAGAATGGATGCACTACGTGACCCTGGGCAAGAAGCTGCAACACCTGATGATGAACGGCCTGGGCCTGGGCTTCATGTCCTCCGGCTACTCCTCCAAGACCGGCAACCCACTGCCTGCATCCCGCCGCATCGATTCCGTGCTGCAAGCAAACGGCGTGGAATCCGGCCCATCCTACTTCTTCGTGGGCGGCCGCGTGTCCGATGAACAGCTGGGCCACGAAGGCATGCGCGAAGATTCCGTGCACATGCGCGGCCCTGCCGAACTGATTCGCGATGATCTGGTGTCCTTCCTGCCTGATTACATGATCCCAAACCGCGTGGTCGTGTTCGAACGCCTGCCACTGTCTGCAAACGGCAAGATCGATGCAAAGGCACTGGCAGCATCCGATCAAGTGAACGCAGAACTGGTGGAACGCCCATTCGTGGCACCACGCACCGAAACCGAAAAGGAAATCGCAGAAGTGTGGGCAAAGTCCCTGCGCCGCGAATCCGTGTCCGTGCAAGATGATTTCTTCGAATCCGGCGGCAACTCCCTGATCGCAGTGGGCCTGATCCGCGAACTGAACTCCCGCCTGGGCGTGTCTCTGCCACTGCAATCCGTCCTGGAATCCCCAACCGTGGAAAAGCTGTCCCGCCGCCTGGAACGCGAAGTGGCACAAGAATCCTCCCGCCTGGTGCGCCTGCACGCAGAAACCGGCAAGGATCGCCCTGTGCTGTGCTGGCCTGGCCTGGGCGGCTACCCAATGAACCTGCGTACCCTGGCCGGCGAAATCGGCCTGGGTCGCTCCTTCTATGGCATTCAAGCACACGGCATCAACGAAGGCGAAGCCCCATACGCAACCATCACCGAAATGGCAAAGGCAGACATCGAAGCAATCAAGGAACTGCAACCAAAGGGCCCATACACCCTGTGGGGCTACTCCTTCGGCGCACGCGTGGCATTCGAAACCGCATATCAGCTGGAACAAGCCGGCGAAAAGGTGGATAACCTGTTCCTGATCGCACCTGGCTCCCCAACTGTCCGCGCCGAAAACGGCAAGGTGTACGGCCGCGAAGCATCCTTCGCAAACCGCGCATACACCACCATCCTGTTCTCCGTGTTCACCGGCACCATCTCCGGCCCTGATCTGGAAAAGTGCCTGGAATCCGCAACCGATGAAGAATCCTTCGCCGGCTTCATCTCCGAACTGAAGGGCATCGATGTGGATCTGGCAAAGCGCATCATCTCCGTGGTGGGTCAGACCTACGAGTTCGAATACTCCTTCCGCGAACTGGCCGAGCGCACCCTGGCAGCACCTGTGACCATCTTCAAGGCACGCGGCGATGATTACTCCTTCATCGAAAACTCCAACGGCTACTCCGCAGAACCACCAACCGTGATCGATCTGGATGCAGATCACTACTCCCTGCTGCGCACCCCTGATATTGGCGAACTGGTGAAGCACATCCGCTACCTGCTGGGCGAACACCACCATCACCACCACTGA |
| Melittin | ATGGGCATCGGTGCCGTGCTTAAAGTCCTCACCACTGGACTGCCAGCATTGATTTCCTGGATCAAGCGCAAGCGTCAGCAACACCATCACCATCACCATTAA |
| Lactoferricin B | ATGTTCAAATGTCGGCGCTGGCAGTGGCGCATGAAGAAGCTGGGCGCACCATCCATCACCTGCGTGCGTCGAGCCTTTCACCATCACCATCACCATTAA |

**^a^** The green bases represent 6×his tag.
